# Sotatercept analog improves cardiopulmonary remodeling and pulmonary hypertension in experimental left heart failure

**DOI:** 10.3389/fcvm.2023.1064290

**Published:** 2023-02-23

**Authors:** Sachindra R. Joshi, Elif Karaca Atabay, Jun Liu, Yan Ding, Steven D. Briscoe, Mark J. Alexander, Patrick Andre, Ravindra Kumar, Gang Li

**Affiliations:** Discovery Group, Acceleron Pharma Inc., a subsidiary of Merck & Co., Inc., Rahway, NJ, United States

**Keywords:** Group 2 pulmonary hypertension (PH), pulmonary arterial hypertension, heart failure with preserved ejection fraction, heart failure with reduced ejection fraction, ActRIIA-Fc, activin, smooth muscle cell, vascular remodeling

## Abstract

Pulmonary hypertension due to left heart disease (PH-LHD) is the most frequent manifestation of PH but lacks any approved treatment. Activin receptor type IIA-Fc fusion protein (ActRIIA-Fc) was found previously to be efficacious in experimental and human pulmonary arterial hypertension (PAH). Here we tested the hypothesis that ActRIIA-Fc improves pulmonary vascular remodeling and alleviates PH in models of PH-LHD, specifically in subtypes of heart failure with reduced ejection fraction (PH-HFrEF) and preserved ejection fraction (PH-HFpEF). Treatment with murine ActRIIA-Fc reduced cardiac remodeling and improved cardiac function in two mouse models of left heart disease without PH, confirming that this inhibitor of activin-class ligand signaling can exert cardioprotective effects in heart failure. In a mouse model of PH-HFrEF with prolonged pressure overload caused by transverse aortic constriction, ActRIIA-Fc treatment significantly reduced pulmonary vascular remodeling, pulmonary fibrosis, and pulmonary hypertension while exerting beneficial structural, functional, and histological effects on both the left and right heart. Additionally, in an obese ZSF1-SU5416 rat model of PH-HFpEF with metabolic dysregulation, therapeutic treatment with ActRIIA-Fc normalized SMAD3 overactivation in pulmonary vascular and perivascular cells, reversed pathologic pulmonary vascular and cardiac remodeling, improved pulmonary and cardiac fibrosis, alleviated PH, and produced marked functional improvements in both cardiac ventricles. Studies *in vitro* revealed that treatment with ActRIIA-Fc prevents an abnormal, glucose-induced, activin-mediated, migratory phenotype in human pulmonary artery smooth muscle cells, providing a mechanism by which ActRIIA-Fc could exert therapeutic effects in experimental PH-HFpEF with metabolic dysregulation. Our results demonstrate that ActRIIA-Fc broadly corrects cardiopulmonary structure and function in experimental PH-LHD, including models of PH-HFrEF and PH-HFpEF, leading to alleviation of PH under diverse pathophysiological conditions. These findings highlight the important pathogenic contributions of activin-class ligands in multiple forms of experimental PH and support ongoing clinical evaluation of human ActRIIA-Fc (sotatercept) in patients with PH-HFpEF.

## Graphical Abstract

In Group 2 pulmonary hypertension (PH), left heart failure can cause retrograde elevation of blood pressure that leads to dysfunction of the pulmonary arterial system and subsequent right heart failure. Activin receptor type IIA-Fc fusion protein (ActRIIA-Fc) improves cardiopulmonary remodeling and function in models of Group 2 PH through mutually reinforcing, beneficial actions at multiple sites. 
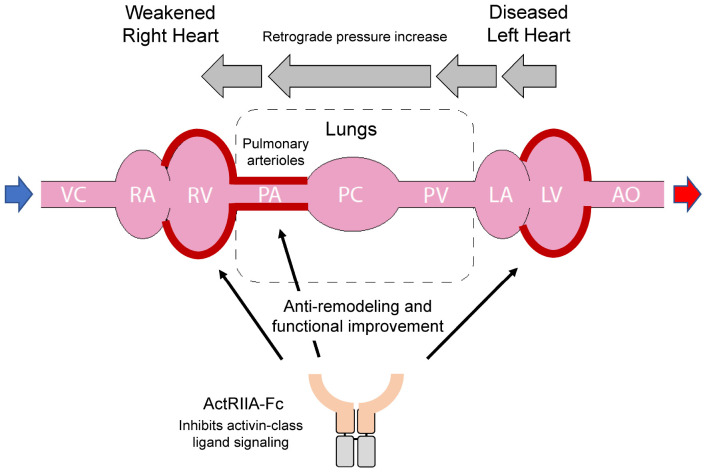

## Introduction

Pulmonary hypertension (PH) accompanies a spectrum of common and rare diseases (1). The most prevalent form, PH associated with left heart disease (PH-LHD, Group 2 PH), is caused by left heart failure, including subtypes with reduced ejection fraction (PH-HFrEF) or preserved ejection fraction (PH-HFpEF) (2, 3). In both subtypes of PH-LHD, functional correlates of pulmonary vascular remodeling predict higher morbidity and mortality than in its absence (4, 5). Cardiac dysfunction associated with HFrEF and HFpEF can be treated with a range of efficacious therapies (6), including the recently-approved empagliflozin (7), but there are no approved treatments for either type of PH-LHD (2). Diverse treatments approved for pulmonary arterial hypertension (PAH, Group 1 PH) have so far been found ineffective in PH-LHD (8).

A key pathogenic factor in PH-LHD is progressive pulmonary vascular remodeling. In left heart disease, elevated pressure can be transmitted in a retrograde manner from the left atrium to the right ventricle (RV) independent of secondary pulmonary vascular remodeling (9). This results in a PH-LHD subtype termed passive, or isolated, post-capillary PH (IpcPH-LHD) (2, 10). A second subgroup of more severely affected patients with precapillary vascular remodeling and combined post- and precapillary PH (CpcPH-LHD) displays pulmonary pathophysiology resembling that in PAH (Group 1 PH) (2, 5, 9, 10). In this case, PH impairs RV function and in the absence of effective treatments eventually causes death by right heart failure. Although features of metabolic syndrome such as higher body mass index, elevated hemoglobin A1c levels, and diabetes are considered risk factors for PH-HFpEF (11–13), the potential influence of metabolic syndrome on pulmonary vascular remodeling in left heart disease is largely unexplored.

Activin receptor signaling is implicated in left heart failure and PAH vascular pathology, raising the possibility that this pathway contributes to PH-LHD pathogenesis. Imbalanced signaling by the transforming growth factor-β (TGF-β) superfamily contributes extensively to pathologic vascular remodeling in PAH, with overactive, pro-proliferative SMAD2/3 signaling occurring along with deficient, antiproliferative SMAD1/5/8 signaling (14). The activin-class ligands activin A, growth differentiation factor 8 (GDF8), and GDF11—prominent activators of SMAD2/3-pathway signaling—are conspicuously upregulated in small pulmonary arteries in both experimental and human PAH (15). Importantly, sequestration of activin-class ligands with an Fc-fusion protein incorporating the extracellular domain of activin receptor type IIA (ActRIIA-Fc) exerts antiproliferative and inflammation-suppressing effects in the lung vasculature, reverses pulmonary vascular remodeling, and reduces PH in experimental PAH, thus exhibiting disease-modifying activity not observed with vasodilator-based therapy (15, 16). Additional evidence implicates activin receptor signaling in pathologic RV remodeling associated with experimental PAH (16) and left heart failure associated with models of systemic pressure overload, aging, ischemia, and acute ischemia-reperfusion injury (17–20). Therapeutic use of ActRIIA-Fc (sotatercept) provides clinically meaningful improvement in patients with PAH, even in those receiving multiple background therapies (21), underscoring the strong pathogenic roles for activin receptor signaling in PAH progression.

Based on these extensive clinical and preclinical observations, we hypothesized that sequestration of activin-class ligands with ActRIIA-Fc will alleviate experimental PH-LHD. Our results in models of PH-HFrEF and PH-HFpEF are the first to indicate that activin receptor signaling plays a critical role in pulmonary vascular remodeling in experimental PH-LHD. The ability of ActRIIA-Fc to reverse pathologic remodeling in the left heart, right heart, and pulmonary vasculature in experimental PH-LHD suggests that multi-ligand sequestration with this agent could be a promising therapeutic approach to treat Group 2 PH.

## Materials and methods

### Animal studies

All animal experiments were approved by the Institutional Animal Care and Use Committee at Acceleron Pharma Inc., a subsidiary of Merck & Co., Inc., Rahway, NJ, USA and performed in accordance with the guidelines from the NIH Guide for the Care and Use of Laboratory Animals. Male C57BL/6 mice (10 weeks old, Jackson Laboratory) were used for TAC and MI models as described (18, 22), and male Balb/c mice (10 weeks old, Jackson Laboratory) were used for the prolonged TAC model to establish PH. Male obese ZSF1 rats (8 and 23 weeks old) and their lean littermates (Charles River, Wilmington, MA, USA) were used for the PH-HFpEF study. PH was established by a single subcutaneous injection of a vascular endothelial growth factor receptor antagonist, Sugen 5416 (SU5416, 100 mg/kg; Cayman), suspended in CMC buffer (0.5% sodium carboxymethyl cellulose, 0.4% polysorbate 80, 0.9% sodium chloride, and 0.9% benzyl alcohol) (23, 24). Animals were euthanized in all experiments by heart and lung removal en bloc according to AVMA guidelines.

### Hemodynamic measurements

Animals were anesthetized with 3–4% isoflurane and placed on controlled heating pads. Right ventricular systolic pressure (RVSP) was measured by advancing a 2F curve tip pressure transducer catheter (SPR-513, Millar Instruments) into the right ventricle (RV) *via* the right jugular vein under 1.5–2% isoflurane anesthesia. En-bloc heart and lungs were collected, and lungs were perfused with physiological saline *via* the right ventricular outflow tract to flush blood cells from the pulmonary circulation. RV hypertrophy was assessed by calculating Fulton’s index, the weight ratio of the RV free wall to the combined left ventricle (LV) + septum [RV/(LV + S)].

### Histopathology and immunohistochemistry

After perfusion, the right bronchus was ligated and the right lung lobes were dissected and snap frozen for biochemical analysis. The left lung lobe was inflated with a formalin-agarose mixture [0.5% w/v low melting agarose in 1% neutral buffered formalin (NBF)] at a constant pressure of 20 cm H_2_O. The inflated lungs were fixed in 10% NBF for 48 h (16). The left lung lobe was blocked, embedded in paraffin, and sectioned. Formalin-fixed, paraffin-embedded lung sections were stained with hematoxylin and eosin (H&E) and Masson’s trichome for histological analysis. Immunohistochemical staining was performed using antibodies against phospho-SMAD3 (pSMAD3) (cat# ab52903, Abcam), activin A (cat# PA5-47004, ThermoFisher), and GDF11 (cat# NBP2-57399, Novus). Dual immunofluorescence staining was performed using combinations of antibodies against pSMAD3 (cat# ab52903, Abcam) and smooth muscle α-actin (cat# A5228, Sigma) or CD31 (cat# ab182981, Abcam). DAPI (4′,6-diamidino-2-phenylindole) was used to identify cell nuclei.

### Morphological analyses

Wall thickness of pulmonary arteries was measured in H&E-stained sections with HALO software. Wall thickness was determined by the formula [outer diameter (OD) – inner diameter (ID)]/OD × 100% as described previously (25). Approximately 30 vessels with OD < 100 μm were randomly selected, and the outer and inner diameters of the vessels were annotated to calculate wall thickness.

### Echocardiography

Animals were anesthetized with 3–4% isoflurane and maintained at 1.5–2% isoflurane during echocardiography. A Vevo 3100 imaging system with MX201 scanhead (VisualSonics, Toronto, ON, Canada) was used to perform echocardiography for measurement of pulmonary. Pulmonary artery acceleration time (PAAT), tricuspid annular plane systolic excursion (TAPSE), isovolumic relaxation time (IVRT), and mitral inflow velocity (E) and mitral annular velocity (E’), from which the E/E’ ratio was derived. Briefly, rats were placed supine on a heated platform and allowed to breathe spontaneously. The right ventricular outflow tract was visualized using a modified parasternal long axis view. PAAT was measured as pulmonary artery blood flow time from start to peak velocity from the pulse wave doppler tracings recorded in the lumen of the main pulmonary artery distal to the pulmonary valve. TAPSE was obtained from the apical four-chamber view directing the M-mode doppler beam through the lateral annulus of the tricuspid valve plane. E, the maximal transmittal flow velocity during early inflow, was recorded using the apical four chamber view. E’, the peak mitral annular velocity during early filling, was obtained by tissue doppler imaging by placing the pulsed waved doppler at the septal corner of the mitral annulus. Using the recordings of transmitral flow velocities, we measured IVRT from the time interval between aortic valve closure and mitral valve opening. For each parameter, measurements from three individual heartbeats per animal were collected and averaged.

### Cell culture

Cardiomyocytes derived from human induced pluripotent stem cells (iPSCs, ATCC-ACS-1021) were cultured as instructed. For cardiomyocyte differentiation *in vitro*, a STEMdiff ventricular cardiomyocyte differentiation kit (STEMCELL Technologies, cat# 05010) was used according to the manufacturer’s instructions. Cardiomyocytes were cultured for 15 days in maintenance medium (STEMCELL Technologies, cat# 05020) to promote maturation. To induce hypertrophy, cardiomyocytes were treated with 10 nM of endothelin 1 (ET-1, Sigma-Aldrich, cat#E7764) for 24 h. Human pulmonary artery smooth muscle cells (PASMCs) were purchased (ATCC, cat# PCS-100-023) and cultured in vascular cell basal medium (ATCC, cat# PCS-100-030) supplemented with a vascular smooth muscle cell growth kit (ATCC, cat# PCS-100-042) in 5% CO_2_ at 37^°^C. Cells from passages 4–7 were used for experiments. Sotagliflozin (Selleckchem, cat# S8103) was used as described for each experiment.

### Western blotting

Cells were lysed in RIPA buffer (Sigma, cat# R0278) on ice for 20 min and centrifuged at 15,000 rpm for 15 min at 4^°^C. Supernatants were quantified by BCA (Thermo Fisher, cat# A53225), and 30–40 μg of protein per sample was used for gel electrophoresis on a 4–15% gel (Bio-Rad, cat# 4568085) and transferred onto nitrocellulose membranes at 250 mA for 90 min. Tissue samples were pulverized and homogenized in RIPA buffer with small pulses for 3 min at 4^°^C. Homogenates were kept on ice for 20 min, and protein quantification was performed by BCA using 20–30 μg of protein per sample. Blots were probed with antibodies against SGLT1 (Cell Signaling Technologies, cat# 5042), pERK1/2 (Cell Signaling Technologies, cat# 9101S), ERK (Cell Signaling Technologies, cat# 4695S), pJNK (Cell Signaling Technologies, cat# 4668S), JNK (Cell Signaling Technologies, cat# 9252S), and GLUT1 (Cell Signaling Technologies, cat# 12939S).

### Real time RT-PCR

Pulverized samples (10–20 mg) were homogenized in QIAzol Lysis Reagent, and total RNA extraction was performed with an RNeasy Plus Mini Kit (Qiagen, cat# 74034) according to the manufacturer’s instructions. RNA concentration was measured with a NanoDrop spectrophotometer (Thermo Fisher Scientific, Waltham, MA, USA), and 0.5–1 μM of RNA was used for cDNA syntheses. Taqman probes (Thermo Fisher Scientific) were used for quantitative real-time RT-PCR (qRT-PCR). At least four technical replicates were used for each group.

### Cellular migration assay

Human PASMCs (5 × 10^4^) were seeded in a Boyden chamber (Sigma-Aldrich, cat# ECM508) in vascular cell basal medium supplemented with a vascular smooth muscle cell growth kit and incubated overnight. The next day, medium in the lower chamber was replaced with basal medium alone or medium containing 25 mM glucose while the upper chamber contained only basal medium. Inhibitors were added to both chambers, but activin A was added only to the lower chamber. Experimental values were measured at the end of a 72 h incubation period.

### Assay for activin A

Human PASMCs (1.5 × 10^5^) were seeded in 24-well plates in vascular cell basal medium supplemented with a vascular smooth muscle cell growth kit. When cells reached 60–70% confluency, they were incubated with glucose-deficient medium for 6 h. Medium was then collected (defined as time zero), and cells were treated for 24 or 48 h with medium containing either 5 or 25 mM glucose. Medium samples were collected at each time point, and activin A concentrations in samples were measured with an ELISA kit (R&D Systems, cat# DAC00B) according to the manufacturer’s instructions.

### Statistical analysis

Data are presented as means ± standard error of the mean (SEM). Comparisons between groups were analyzed using either Student’s *t*-test or ANOVA with Dunnett’s or Tukey’s *post-hoc* test for multiple comparisons. Differences were considered significant at *P* < 0.05.

## Results

### ActRIIA-Fc improves cardiac structure, function, and histology and reduces cardiomyocyte injury markers in models of left heart failure

**FIGURE 1:**
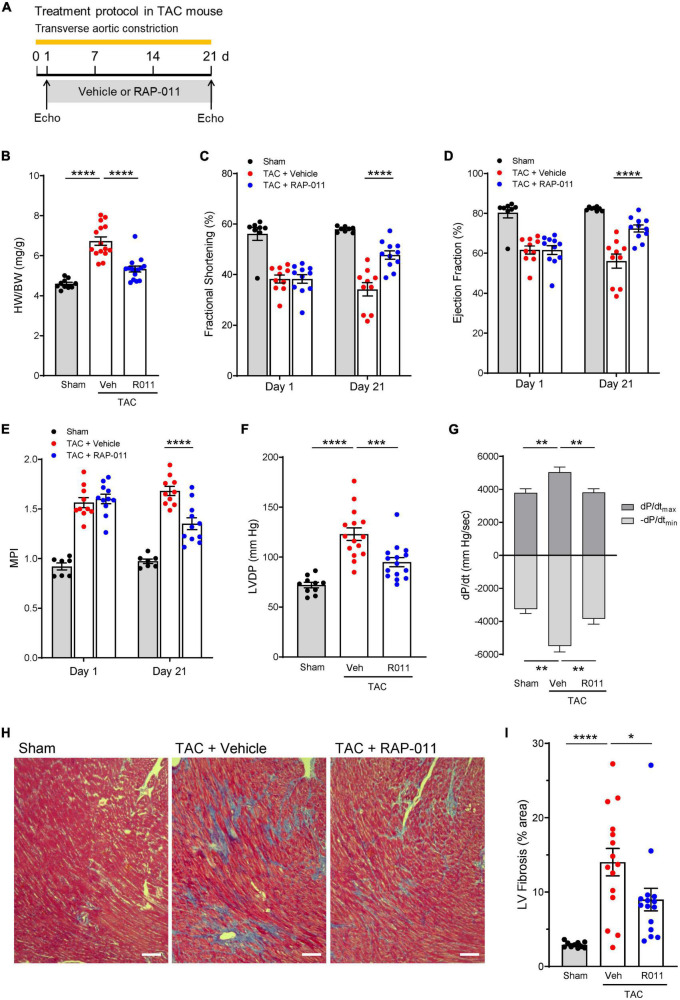
ActRIIA-Fc exerts structural, functional, and anti-fibrotic cardioprotective effects in a transverse aortic constriction (TAC) mouse model of left heart failure caused by sustained pressure overload. **(A)** Experimental approach used to assess effects of ActRIIA-Fc (RAP-011). Wild-type mice were subjected to TAC and treated twice weekly with RAP-011 (R011, 10 mg/kg, s.c.) or vehicle (veh, phosphate-buffered saline, PBS) for 3 weeks starting one day post-surgery. **(B)** Heart weight normalized to body weight (HW/BW), **(C)** fractional shortening, **(D)** ejection fraction, **(E)** myocardial performance index (MPI), **(F)** LV developed pressure (LVDP), and **(G)** peak rates of LV pressure rise (dP/dt_*max*_) and decline (-dP/dt_*min*_). Data are means ± SEM (*n* = 10–15 mice per group for day 21). **(H)** Representative images of LV sections stained with Masson’s trichrome blue to detect fibrosis (scale bar, 100 μm), and **(I)** quantification of percentage area occupied by fibrotic tissue. Data are means ± SEM (*n* = 10–15 mice per group). Analysis by one-way ANOVA and Dunnett’s *post-hoc* test (*P < 0.05, **P < 0.01, ***P < 0.001, ****P < 0.0001).

We evaluated ActRIIA-Fc effects in mouse models of left heart failure caused by sustained pressure overload or myocardial infarction because activin receptor signaling has been implicated in multiple forms of left heart disease (17–20). LV pressure overload induced in mice by transverse aortic constriction (TAC) for 3 weeks led to detrimental changes in cardiac structure, function, and histology (Figure 1). Treatment with a murine form of ActRIIA-Fc (RAP-011) exerted significant cardioprotective effects on these parameters when compared with vehicle treatment (Figure 1). This result is consistent with previously reported beneficial effects of activin receptor-like kinase 4 (ALK4) haploinsufficiency on cardiac hypertrophy, dysfunction, and fibrosis in a similar mouse model of LV pressure overload (17). Treatment with RAP-011 also produced beneficial effects in a mouse model of myocardial infarction (Supplementary Figure 1), consistent with previously reported benefits of ActRIIB ligand inhibition under comparable conditions (20). These results confirm cardioprotective effects of ActRIIA-Fc similar to those observed with other methods of activin-signaling inhibition in experimental heart failure.

To determine whether ActRIIA-Fc exerts cardioprotective effects through regulation of cardiomyocyte signaling, we evaluated effects of a human form of ActRIIA-Fc (ACE-011) on injury markers in cultured cardiomyocytes derived from human induced pluripotent stem cells (iPSCs) (26). Treatment with endothelin-1 (ET-1) induced cellular injury in these cardiomyocytes, including increased *NPPB* expression and a shift in the relative abundance of myosin heavy chain isoforms from α to β (increased *MYH7*:*MYH6* ratio) (Supplementary Figure 2). Co-treatment with ACE-011 significantly limited ET-1–induced *NPPB* expression and partially normalized the *MYH7*:*MYH6* ratio in these cells (Supplementary Figure 2). Together, the above results indicate that ActRIIA-Fc exerts cardioprotective effects in multiple mouse models of left heart failure, consistent with previous studies of activin signaling pathway inhibition, and imply that activin-class ligands act directly on cardiomyocytes to drive pathogenic cellular activity in these contexts.

### ActRIIA-Fc improves cardiopulmonary function and alleviates PH in a mouse model of PH-HFrEF

**FIGURE 2:**
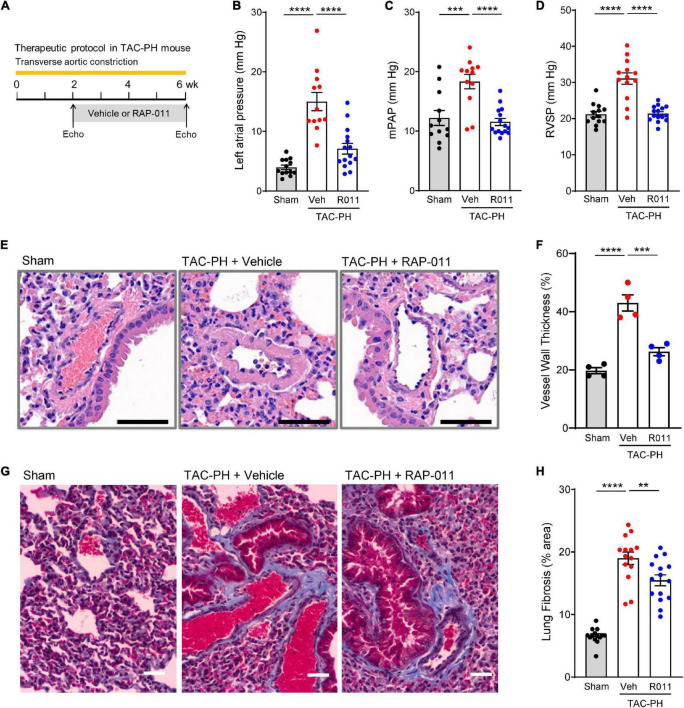
ActRIIA-Fc improves pulmonary vascular remodeling and reduces PH in a TAC-PH mouse model. **(A)** Experimental approach used to assess effects of ActRIIA-Fc (RAP-011). Wild-type mice were subjected to TAC and treated twice weekly with RAP-011 (R011, 10 mg/kg, s.c.) or vehicle (veh, PBS) for 4 weeks starting 2 weeks post-surgery to promote development of PH in the untreated state. **(B)** Left atrial pressure, **(C)** mean pulmonary arterial pressure (mPAP), and **(D)** RV systolic pressure (RVSP). **(E)** Images of representative lung sections stained with hematoxylin and eosin showing degree of vascular remodeling. Scale bar, 50 μm. **(F)** Quantification of vessel wall thickness as a percentage of vessel outer diameter. **(G)** Images of lung sections stained with Masson’s trichrome to detect fibrosis. Scale bar, 50 μm. **(H)** Quantification of fibrotic tissue area. Data are means ± SEM. Analysis by one-way ANOVA and Dunnett’s *post-hoc* test (**P < 0.01, ***P < 0.001, ****P < 0.0001).

To determine whether ActRIIA-Fc alleviates PH caused by LHD, we evaluated effects of RAP-011 treatment in a TAC-PH mouse model of PH-HFrEF in which prolonged TAC causes PH (Figure 2A) (27). Compared with sham controls, this extended TAC intervention produced elevated pressures in the left atrium and induced PH (Figures 2B–D), hemodynamic effects associated with pathologic pulmonary vascular remodeling (Figures 2E, F) and pulmonary fibrosis (Figures 2G, H). Treatment with RAP-011 beginning 2 weeks after the start of TAC significantly improved each of these cardiopulmonary parameters as compared with vehicle treatment (Figures 2B–H). These results indicate that RAP-011 alleviates PH arising from sustained LV pressure overload.

**FIGURE 3:**
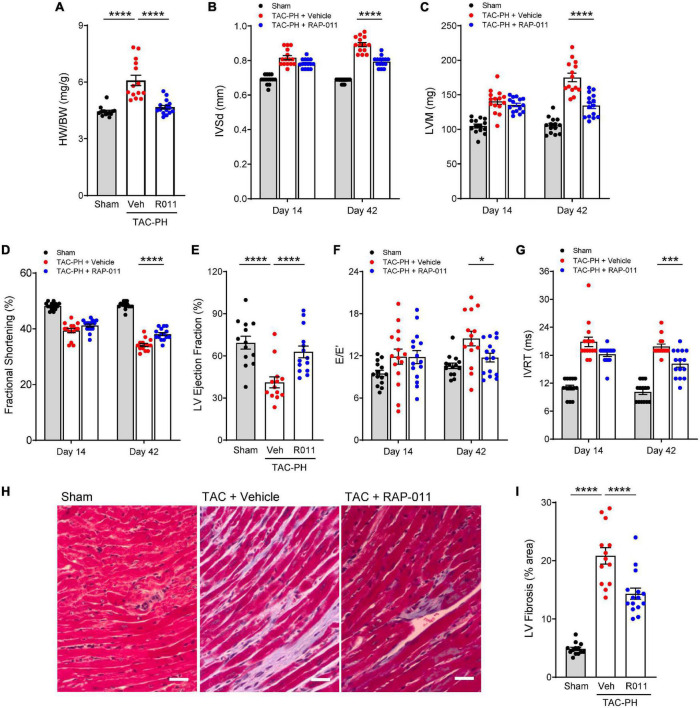
ActRIIA-Fc reduces left ventricle (LV) remodeling and improves left heart function in the TAC-PH mouse model. **(A)** Heart weight normalized to body weight (HW/BW), **(B)** interventricular septum thickness in diastole (IVSd), **(C)** LV mass (LVM), **(D)** fractional shortening, **(E)** LV ejection fraction, **(F)** mitral inflow velocity (E) and mitral annular velocity (E’) ratio (E/E’), and **(G)** isovolumic relaxation time (IVRT). Data are means ± SEM (*n* = 10–15 mice per group for day 42). **(H)** Representative images of LV sections stained with Masson’s trichrome blue to detect fibrosis (scale bar, 50 μm) and **(I)** quantification of percentage area occupied by fibrotic tissue. Data are means ± SEM (*n* = 10–15 mice per group). Analysis by one-way ANOVA and Dunnett’s *post-hoc* test (*P < 0.05, ***P < 0.001, ****P < 0.0001). RAP-011 (R011), vehicle (veh, PBS).

We then investigated whether alleviation of PH in this model by treatment with RAP-011 was accompanied by beneficial effects on heart structure. Treatment with RAP-011 biweekly for 6 weeks beginning 2 weeks after TAC onset provided significant protection against adverse cardiac remodeling when compared with vehicle-treated mice (Figure 3). Specifically, RAP-011 treatment reduced cardiac hypertrophy (Figures 3A–C), improved LV contractility (Figures 3D, E) and LV diastolic function (Figures 3F, G), and reduced LV fibrosis (Figures 3H, I) compared with these measures in vehicle-treated controls. RAP-011 treatment also conferred significant protection against RV remodeling in this model (Supplementary Figure 3). Together, these findings establish that treatment with ActRIIA-Fc alleviates PH and protects against pathologic cardiopulmonary remodeling in this model of PH-HFrEF.

### ActRIIA-Fc reduces pulmonary remodeling and improves cardiopulmonary function in the obese ZSF1-Su rat model of PH-HFpEF

We next tested whether therapeutic treatment with ActRIIA-Fc would exert favorable cardiopulmonary effects in a rat model of PH-HFpEF. In this two-hit model (23), obese ZSF1 rats develop PH-HFpEF after a single high dose of SU5416 (Su), an inhibitor of vascular endothelial growth factor receptor-2 used to induce pulmonary endothelial injury. Treatment with SU5416 alone did not induce pulmonary hypertension or diastolic dysfunction in lean rats (Supplementary Figure 4), while ZSF1 rats in the absence of SU5416 exhibited diastolic dysfunction without pulmonary hypertension (Supplementary Figure 4). Obese ZSF1-Su rats have been shown to recapitulate hemodynamic features and clinical outcomes of patients with PH-HFpEF, notably including CpcPH-HFpEF. As one aspect of our evaluation in this model, we compared ActRIIA-Fc effects with those of sildenafil, a representative phosphodiesterase type 5 (PDE5) inhibitor, because therapeutic agents in this class have been assessed in patients with HFpEF (28, 29). Preclinically, sildenafil therapy produces modest improvements in systemic hypertension and LV stiffness in obese ZSF1 rats without PH (30), which model disease features observed in some patients with HFpEF.

**FIGURE 4:**
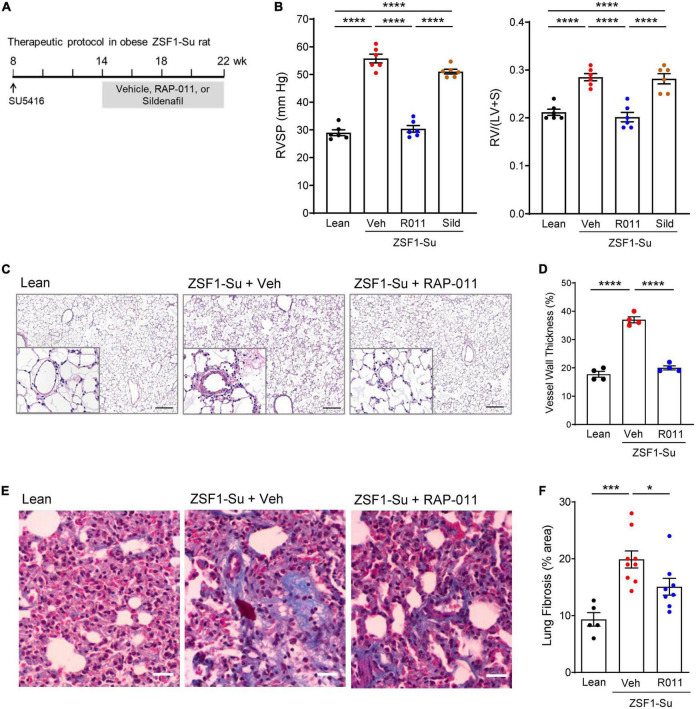
Therapeutic treatment with ActRIIA-Fc improves pulmonary remodeling and cardiopulmonary function in the obese ZSF1-Su rat model of PH-HFpEF. **(A)** Experimental approach used to evaluate therapeutic effects of ActRIIA-Fc (RAP-011, R011) in an obese ZSF1-Su rat model of PH-HFpEF. Rats were treated at 8 weeks of age with a single dose of SU5416 (100 mg/kg, s.c). After allowing 6 weeks for development of PH, rats were treated with RAP-011 (10 mg/kg, s.c., twice weekly), sildenafil (30 mg/kg, p.o., twice daily), or vehicle (Veh, PBS) for 8 weeks. **(B)** Effects of RAP-011 (R011) or sildenafil (Sild) on RV systolic pressure (RVSP) and Fulton index [RV/(LV + S)], a measure of RV hypertrophy. **(C)** Images of representative lung sections stained with hematoxylin and eosin, with insets showing degree of vascular remodeling. Scale bar, 200 μm. **(D)** Vessel wall thickness as a percentage of vessel outer diameter. **(E)** Images of lung sections stained with Masson’s trichrome to detect fibrosis. Scale bar, 50 μm. **(F)** Quantification of fibrotic tissue area. Data are means ± SEM. Analysis by one-way ANOVA and Tukey’s **(B)** or Dunnett’s **(D, F)** *post-hoc* test. *P < 0.05; ***P < 0.001; ****P < 0.0001. Lean, lean control rats.

To mimic a clinical stage in which disease has progressed substantially before onset of treatment, we initiated treatment with either RAP-011 or vehicle 6 weeks after administration of SU5416, at which time RV dysfunction is prominent, and continued treatment biweekly for 8 weeks (Figure 4A). As determined by right heart catheterization and echocardiography, obese ZSF1-Su rats treated with vehicle exhibited significantly increased RV systolic pressure (RVSP) and reduced pulmonary artery acceleration time (PAAT) (Figure 4B and Supplementary Figure 5). Therapeutic treatment with RAP-011 fully reversed these changes to values observed in lean controls, whereas sildenafil treatment improved RVSP by 19% and PAAT by 36%. In addition, treatment with RAP-011, but not sildenafil, fully normalized RV hypertrophy (Fulton index) and tricuspid annular plane systolic excursion (TAPSE) (Figure 4B and Supplementary Figure 5). Histologic analysis confirmed the presence of pulmonary vascular remodeling in obese ZSF1-Su rats, as described previously. Therapeutic treatment with RAP-011 restored pulmonary vessel structure to conditions observed in lean controls (Figures 4C, D). Finally, elevated levels of pulmonary fibrosis present in obese ZSF1-Su rats were partially reversed by therapeutic treatment with RAP-011 (Figures 4E, F). Together, these results indicate that therapeutic treatment with ActRIIA-Fc reduces fibrosis, reverses RV and pulmonary vascular remodeling, improves RV function, and alleviates PH in the obese ZSF1-Su model of PH-HFpEF.

### ActRIIA-Fc improves remodeling and function of both left and right heart in obese ZSF1-Su rats

Based on the beneficial cardiac effects of ActRIIA-Fc observed in the above experiments, we investigated cardiac effects of ActRIIA-Fc treatment more extensively in the PH-HFpEF model. As before, we initiated treatment with either ActRIIA-Fc (RAP-011) or vehicle 6 weeks after administration of SU5416 and continued treatment biweekly for 8 weeks (weeks 14–22, Figure 4A).

**FIGURE 5:**
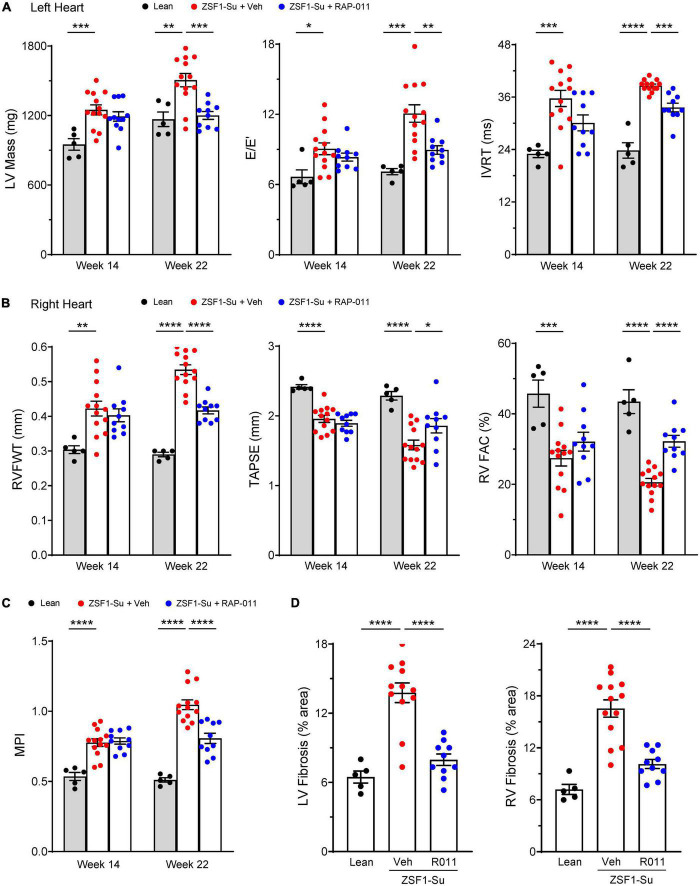
ActRIIA-Fc improves remodeling and function of both left and right heart in obese ZSF1-Su rats. Effects of ActRIIA-Fc (RAP-011, R011) on **(A)** left heart parameters LV mass, mitral inflow velocity (E) and mitral annular velocity (E’) ratio (E/E’), and isovolumic relaxation time (IVRT); **(B)** right heart parameters RV free-wall thickness (RVFWT), tricuspid annular plane systolic excursion (TAPSE), and RV fractional area change (RVFAC); **(C)** myocardial performance index (MPI); and **(D)** quantification of fibrosis in LV and RV. Data are means ± SEM (*n* = 5–13 rats per group). Analysis by one-way ANOVA and Dunnett’s *post-hoc* test. *P < 0.05; **P < 0.01; ***P < 0.001; ****P < 0.0001.

As determined by right heart catheterization and echocardiography, LV and RV dysfunction and abnormal remodeling were prominent in diseased rats before treatment initiation at week 14 (Figures 5A–C). Follow-up echocardiographic analysis at week 22 revealed further disease progression of all evaluated parameters in vehicle-treated, obese ZSF1-Su rats, compared with assessments made either at baseline (week 14) or in lean controls (week 22) (Figures 5A–C). Therapeutic treatment of obese ZSF1-Su rats with RAP-011 normalized LV mass and produced significant improvements in two measures of LV diastolic dysfunction, E/E’ [the ratio of mitral inflow velocity (E) to mitral annular velocity (E’)] and isovolumic relaxation time (IVRT) (Figure 5A). RAP-011 treatment also significantly improved RV remodeling [RV free-wall thickness (RVFWT)] and RV function [RV fractional area change (RVFAC) and TAPSE] (Figure 5B), which confirmed the result for TAPSE above (Supplementary Figure 5). In addition, RAP-011 treatment in obese ZSF1-Su rats significantly improved the myocardial performance index (MPI), a measure of global cardiac function (Figure 5C). Finally, RAP-011 treatment significantly reduced fibrosis in both the LV and RV compared with vehicle-treated controls (Figure 5D and Supplementary Figure 6). These results provide compelling evidence that beneficial effects of ActRIIA-Fc treatment on PH are accompanied by reversal of pathologic cardiac remodeling as well as functional improvements in both ventricles in this model of PH-HFpEF.

### ActRIIA-Fc inhibits canonical and non-canonical TGF-β superfamily signaling in the lungs of obese ZSF1-Su rats

**FIGURE 6:**
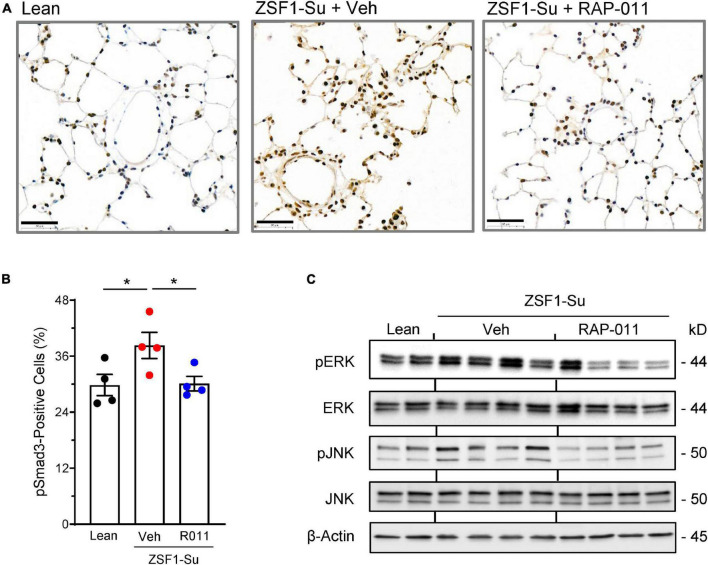
ActRIIA-Fc inhibits canonical and non-canonical signaling associated with the TGF-β superfamily pathway in the lung of obese ZSF1-Su rats. **(A)** Representative images of lung sections immunostained for phospho-Smad3 (pSmad3) in obese ZSF1-Su rats and lean controls after treatment with ActRIIA-Fc (RAP-011, R011) or vehicle (veh, PBS) as in Figure 1A. Scale bar, 50 μm. **(B)** Percentage of pSmad3-positive cells in lungs based on assessment of 30 high-magnification fields per rat. **(C)** Lung homogenates immunoblotted for phospho–extracellular-signal-regulated kinase (pERK), total ERK, phospho-c-Jun N-terminal kinase (pJNK), total JNK, and β-actin. Data are means ± SEM. Analysis by one-way ANOVA and Dunnett’s *post-hoc* test. *P < 0.05.

We next investigated signaling pathways whose activity is influenced by ActRIIA-Fc in the lungs of obese ZSF1-Su rats. Compared with lean controls, obese ZSF1-Su rats displayed elevated activation of SMAD3 in pulmonary perivascular cells as determined by immunostaining for phospho-SMAD3 (pSMAD3) (Figures 6A, B). In obese ZSF1-Su rats, therapeutic treatment with RAP-011 normalized pSMAD3 immunostaining in these perivascular cells (Figures 6A, B), which were shown separately by dual immunofluorescence staining to include vascular smooth muscle cells and endothelial cells (Supplementary Figures 7, 8). Consistent with disease-related overactivation of SMAD3 in the pulmonary vasculature, perivascular regions of ZSF-Su rat lung were found to contain increased immunostaining for activin A and GDF11 (Supplementary Figure 9), prominent activators of SMAD2/3 signaling previously implicated in PAH (15). Obese ZSF1-Su rats treated with vehicle exhibited elevated activation of the p38 mitogen-activated protein kinase (MAPK) and c-Jun N-terminal kinase (JNK) pathways in lung as determined by protein blot analysis of phospho–extracellular-signal-regulated kinase (pERK) and pJNK levels in whole lung lysate (Figure 6C). In obese ZSF1-Su rats, RAP-011 treatment reduced pERK and pJNK levels, whereas total ERK and total JNK expression remained unchanged (Figure 6C). These results indicate that lung tissue in obese ZSF1-Su rats displays elevated activation of pathways associated with TGF-β superfamily canonical (pSMAD3) and non-canonical (pERK and pJNK) signaling and that therapeutic treatment with ActRIIA-Fc inhibits the activation of both types.

### ActRIIA-Fc inhibits glucose-induced release of activin and migration of human PASMCs mediated by the SGLT pathway

**FIGURE 7:**
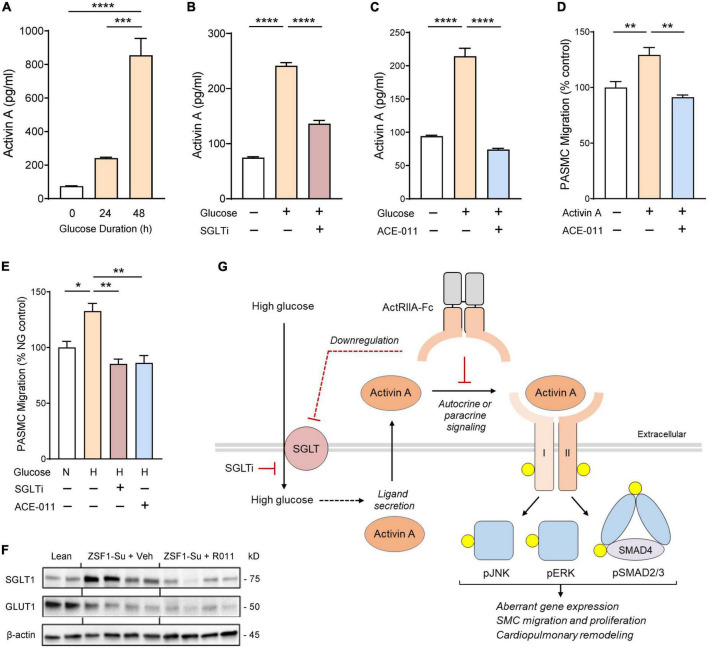
ActRIIA-Fc inhibits glucose-induced release of activin A and migration by human pulmonary artery smooth muscle cells (PASMCs) mediated through the SGLT pathway. **(A)** Glucose-induced activin A release by hPASMCs. **(B)** Effect of SGLT inhibitor (SGLTi) sotagliflozin on glucose-induced release of activin A by hPASMCs. **(C)** Effect of human ActRIIA-Fc (ACE-011) on glucose-induced release of activin A by hPASMCs. **(D)** ACE-011 inhibits activin A-induced migration of hPASMCs. **(E)** Effects of SGLTi or ACE-011 on glucose-induced migration of hPASMCs. N or NG, normal glucose concentration (5 mM); H or HG, high glucose concentration (25 mM). Data are means ± SEM. Analysis by one-way ANOVA and Dunnett’s *post-hoc* test. *P < 0.05; **P < 0.01; ***P < 0.001; ****P < 0.0001. **(F)** Effect of RAP-011 (R011) on dysregulated expression of glucose transporters in lung of obese ZSF1-Su rats. Lean, lean control rats; veh, vehicle (PBS). **(G)** Schematic overview of proposed mechanisms by which ActRIIA-Fc normalizes aberrant PASMC activity induced by high glucose levels in the obese ZSF1-Su rat. For clarity, activin A and its receptors are depicted as monomeric, and the non-covalent complex between mature activin A and its prodomain is not shown. Yellow circles identify phosphorylated proteins and dashed lines indicate mechanisms not yet delineated. I, activin receptor-like kinase 4 or 7; II, ActRIIA or ActRIIB; SGLT, sodium-glucose cotransporter; SGLTi, SGLT inhibitor.

To explore mechanisms by which ActRIIA-Fc improves pulmonary vascular remodeling in obese ZSF1-Su rats, we examined the activity of hPASMCs *in vitro* under elevated glucose conditions to model metabolic comorbidities observed in patients with PH-HFpEF. We observed that glucose treatment produced a significant, time-dependent increase in activin A release by hPASMCs, with pronounced increases in extracellular activin A concentrations occurring at 24 h of exposure and increases of more than 10-fold at 48 h (Figure 7A). Sotagliflozin, a dual inhibitor of sodium-glucose cotransporters (SGLT), significantly reduced activin A release by hPASMCs under elevated glucose conditions (Figure 7B), implicating this transporter type in glucose-induced release of activin A. Treatment of cultured hPASMCs with ACE-011 completely blocked glucose-induced release of activin A (Figure 7C).

We next investigated migratory activity of hPASMCs *in vitro* using a Boyden chamber migration assay. Treatment with activin A significantly stimulated migration of hPASMCs, and this migratory effect was blocked completely by co-treatment with ACE-011 (Figure 7D). Treatment with elevated glucose levels increased migration of hPASMCs beyond rates observed with normal physiologic glucose levels, and this effect was completely blocked by either SGLT inhibitor or ACE-011 (Figure 7E). These results implicate a novel mechanism contributing to aberrant vascular remodeling in which glucose-induced release of activin A by PASMCs triggers their transition to an abnormal migratory phenotype, thereby promoting maladaptive pulmonary vascular modeling in the context of metabolic disorder.

Finally, we explored whether SGLT is similarly dysregulated in lungs of obese ZSF1-Su rats *in vivo*. Compared with lean controls, obese ZSF1-Su rats treated with vehicle exhibited increased pulmonary expression of SGLT1 protein and reduced expression of glucose transporter GLUT1 (Figure 7F). Treatment of obese ZSF1-Su rats with RAP-011 normalized SGLT1 protein expression in lung tissue without detectable changes in GLUT1 protein expression (Figure 7F). As summarized schematically in Figure 7G, our concordant findings from *in vitro* and *in vivo* models implicate SGLT, activin A, and activin receptor-mediated signaling—both canonical and non-canonical—as key components in a PASMC-mediated process by which hyperglycemia promotes aberrant pulmonary vascular remodeling and PH in PH-HFpEF. These results also provide mechanistic insight into ActRIIA-Fc alleviation of PH associated with metabolic comorbidities in this preclinical model of HFpEF.

## Discussion

There is major unmet need for patients with PH-LHD. PH is highly prevalent in patients with LHD and is associated with a worse prognosis than LHD without PH (2, 5). Elevated left atrial pressure due to LHD causes pulmonary venous congestion and increased hydrostatic pressure in pulmonary capillaries, thereby leading to IpcPH-LHD and CpcPH-LHD (2). Despite shared features in the pathophysiology of CpcPH-LHD and PAH (11), including pre-capillary pulmonary vascular remodeling, vasodilators used to treat PAH are ineffective in patients with PH-LHD and can even be harmful (8, 31). In the present study, we find that therapeutic treatment with ActRIIA-Fc exerts reverse remodeling effects in the pulmonary vasculature, left heart, and right heart in rats with experimental CpcPH-HFpEF, whereas the vasodilator sildenafil does not, consistent with previous clinical results (32). Here we also find that ActRIIA-Fc exerts protective cardiopulmonary effects in an established model of a second major type of PH-LHD, CpcPH-HFrEF.

Pulmonary vascular remodeling is a key pathologic feature in patients with PH and is associated with diminished pulmonary arterial compliance, impaired RV–pulmonary arterial coupling, reduced diffusing capacity of the lungs, abnormal RV remodeling, and higher patient morbidity or mortality (2, 9, 10, 33). We recently identified unbalanced signaling by the SMAD pathways—with important contributions by activin-class ligands—in pathogenic vascular remodeling in lung that underlies human and experimental PAH (15). In the present study, we similarly found evidence of increased expression of activin A and GDF11 in the pulmonary vasculature in two experimental models of PH-LHD. Several cell types are implicated in pulmonary vascular remodeling, including endothelial cells, vascular SMCs (VSMCs), adventitial fibroblasts, and immune cells (15, 16, 34). Here we focused our analysis *in vitro* on pulmonary VSMCs because they are a nearly universal component of vascular remodeling (35) and contribute to progressive pre-capillary remodeling in CpcPH-HFpEF (3, 9), as confirmed in our histological assessment of obese ZSF1-Su rats. Unlike terminally differentiated skeletal or cardiac muscle cells, VSMCs retain remarkable plasticity in adulthood, which enables vessel growth and adaptive remodeling but also contributes broadly to cardiovascular pathologies (36).

The lung has historically not been considered an organ prominently afflicted by glucose dysregulation in diabetes (37). However, our studies using hPASMCs reveal an activin-mediated mechanism by which hyperglycemia causes an abnormal phenotypic shift in hPASMCs that could contribute to pulmonary vascular remodeling and PH in experimental PH-HFpEF. Despite the resemblance between CpcPH-HFpEF and PAH with regard to vascular remodeling, the glucose-associated aspect of this mechanism differs from other mechanistic elements identified so far for activin signaling and pulmonary vascular remodeling in the context of experimental PAH (15, 16, 38).

In our model, activin A produced by hPASMCs acts in an autocrine or paracrine manner to mediate a glucose-inducible shift in these cells to an abnormal migratory phenotype (Figure 7). We speculate that activin A secreted by PASMCs *in vivo* could potentially also affect the activity of other cell types in the pulmonary vascular microenvironment. Our results indicate that glucose induction of activin A release by hPASMCs is partly mediated by SGLT. Additionally, our results indicate that SGLT1 protein is upregulated in the lungs of obese ZSF1-Su rats in the setting of metabolic syndrome, and ActRIIA-Fc treatment normalizes these SGLT1 levels. Thus, beneficial effects of ActRIIA-Fc treatment on PASMCs under these conditions arise not only from sequestration of extracellular activin A and potentially other activin-class ligands—with consequent inhibition of canonical and non-canonical activin receptor-mediated signaling—but also from downregulation of SGLT1 protein by an indirect mechanism yet to be determined. Although many studies have demonstrated adverse effects of hyperglycemia on VSMC or endothelial cell function (39), surprisingly few have examined the severity of PH in patients with diabetes, especially for PH types other than PAH (40). However, clinical studies have identified activin A as a prominent marker of cardiovascular pathology, including increased arterial intima–media thickness, in the context of metabolic syndrome (41–44).

In the present study, ActRIIA-Fc also exerted protective cardiopulmonary effects in experimental PH-HFrEF. As underscored here by the beneficial effects of ActRIIA-Fc in models of left heart disease without PH, activin receptor signaling has been implicated previously as an important contributor to pathologic cardiac remodeling in multiple types of left heart failure. This aspect of ActRIIA-Fc activity resembles cardioprotective effects of ALK4 haplodeficiency and effects of other activin receptor pathway inhibitors (ActRIIB-Fc and a dual-specific antibody against ActRIIA and ActRIIB) in models of left ventricular failure associated with aging or systemic pressure overload (17, 18). The roles of activin pathway signaling in cardiac muscle, especially by activin A and GDF11, have been controversial (18). However, recent studies consistently implicate activin receptor–mediated SMAD2/3 signaling as a partially compensatory pathway that becomes maladaptive in heart failure and ischemia-reperfusion injury (17–20). Elevated levels of circulating activin A in patients support a role for this ligand in abnormal myocardial remodeling, diabetic cardiomyopathy, HFrEF, and HFpEF (42, 44–46). Interestingly, activin A, GDF8, and GDF11 induce similar but non-identical pathologic profiles in left ventricular cardiomyocytes (18). We speculate that multi-ligand inhibition might therefore advantageously prevent overlapping as well as distinct activities of activin-class ligands, which could be mutually reinforcing in the therapeutic treatment of cardiovascular disease.

Our results obtained with complementary experimental models display robust concordance and support the translatability of our findings. In this study, we focused on pulmonary VSMCs due in part to their important roles in vascular muscularization and remodeling. However, evidence strongly suggests that other pulmonary vascular cell types contribute to PH and are regulated extensively by activin pathway signaling (14–16, 38). It will therefore be important to investigate the contributions of these signaling mechanisms to dysregulated endothelial cell, fibroblast, and immune cell activities implicated in PH-HFpEF. Immune cells and inflammatory processes play major roles in PH (14, 47–49), and recent evidence indicates that ActRIIA-Fc suppresses inflammation as one component of its multi-factorial mechanism of action in experimental PAH (16). An ongoing phase 2 study is evaluating sotatercept, a human analog of ActRIIA-Fc, in patients with CpcPH-HFpEF.

## Data availability statement

The original contributions presented in this study are included in this article/Supplementary material, further inquiries can be directed to the corresponding author.

## Ethics statement

The animal study was reviewed and approved by the Institutional Animal Care and Use Committee at Acceleron Pharma Inc., a subsidiary of Merck & Co., Inc., Rahway, NJ, USA and performed in accordance with the guidelines from the NIH Guide for the Care and Use of Laboratory Animals.

## Author contributions

SJ, EA, and GL planned the research. SJ and JL performed *in vivo* experiments. EA, JL, and YD performed *in vitro* experiments. SJ, EA, JL, YD, and GL analyzed data. PA, RK, and GL provided guidance on experimental designs and data analysis. SJ, EA, SB, MA, and GL wrote the manuscript, which was reviewed and approved for submission by all authors.
