# Supplementary material for: Sotatercept analog improves cardiopulmonary remodeling and pulmonary hypertension in experimental left heart failure
Source: Front Cardiovasc Med. 2023 Feb 23;10:1064290. doi: 10.3389/fcvm.2023.1064290 (PMC9996114; doi:10.3389/fcvm.2023.1064290)
Supplement: Supplementary file 1 [file Data_Sheet_1.pdf]

Supplementary Material

Sotatercept analog improves cardiopulmonary remodeling and pulmonary hypertension in experimental left heart failure

Sachindra R. Joshi, Elif Karaca Atabay, Jun Liu, Yan Ding, Steven D. Briscoe, Mark J. Alexander, Patrick Andre, Ravindra Kumar, Gang Li

Suppl. Fig. 1. ActRIIA-Fc reduces LV remodeling and improves cardiac function in a mouse model of myocardial infarction

Suppl. Fig. 2. ActRIIA-Fc reduces cellular injury in cardiomyocytes derived from human induced pluripotent stem cells

Suppl. Fig. 3. ActRIIA-Fc reduces RV remodeling and improves right heart function in a TAC-PH mouse model

Suppl. Fig. 4. A two-hit model of PH-HFpEF induced by SU5416 in obese ZSF1 rats but not lean rats

Suppl. Fig. 5. Therapeutic treatment with ActRIIA-Fc improves right heart function more effectively than sildenafil in obese ZSF1-Su rats

Suppl. Fig. 6. ActRIIA-Fc reduces fibrosis in the left and right ventricles in obese ZSF1-Su rats

Suppl. Fig. 7. ActRIIA-Fc reduces SMAD3 overactivation in pulmonary vascular smooth muscle cells of obese ZSF1-Su rats

Suppl. Fig. 8. ActRIIA-Fc reduces SMAD3 overactivation in pulmonary endothelial cells of obese ZSF1-Su rats

Suppl. Fig. 9. Increased expression of activin A and GDF11 in the pulmonary vasculature of PH-LHD models

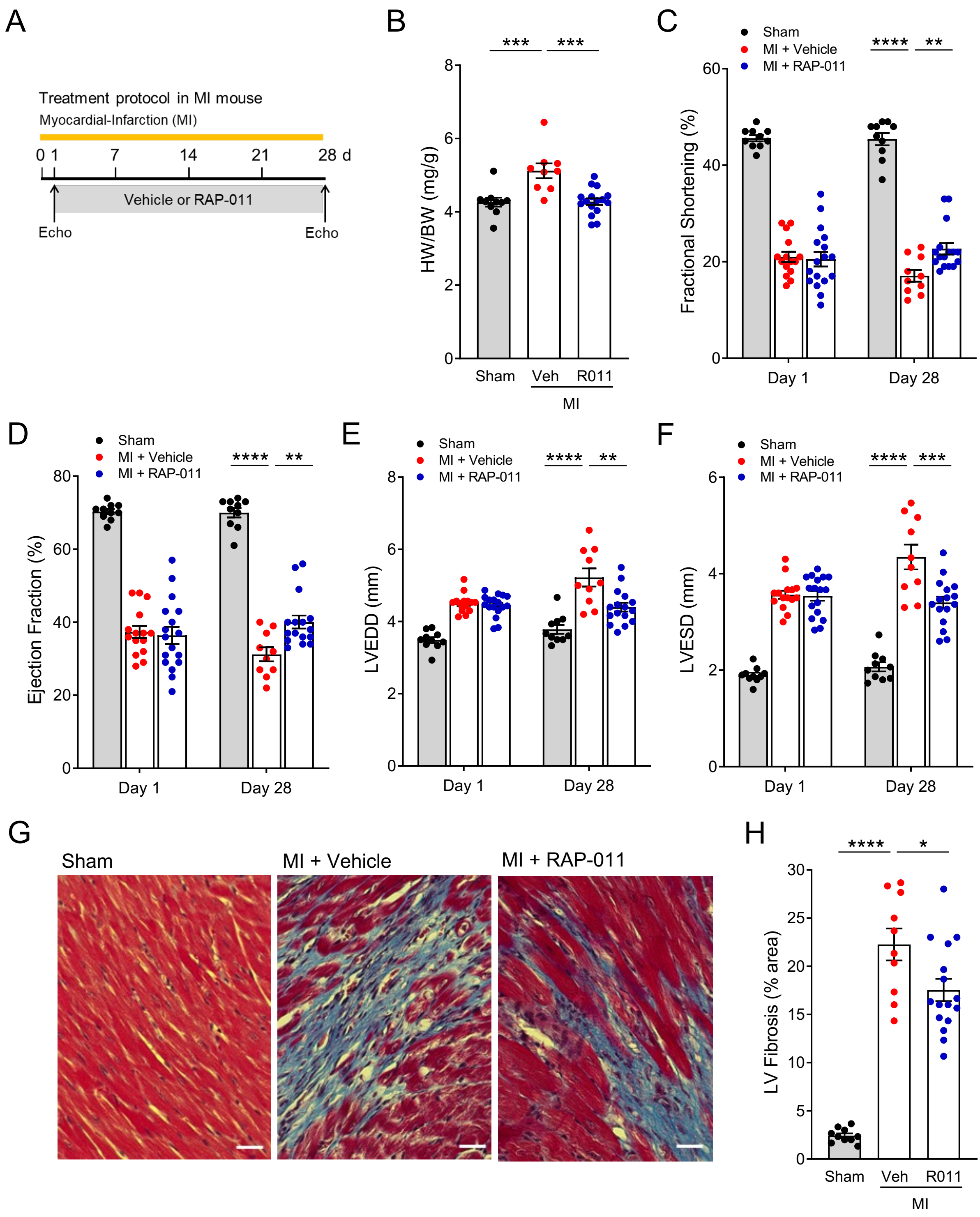

Supplementary Figure 1. ActRIIA-Fc reduces LV remodeling and improves cardiac function in a mouse model of myocardial infarction. (**A**) Experimental approach used to assess effects of ActRIIA-Fc (RAP-011). Wild-type mice were subjected to myocardial infarction (MI) and treated twice weekly with RAP-011 (R011, 10 mg/kg, s.c.) or vehicle (veh, phosphate-buffered saline, PBS) for 4 weeks starting one day post infarct. (**B**) Heart weight normalized to body weight (HW/BW), (**C**) fractional shortening, (**D**) ejection fraction, (**E**) LV end-diastolic diameter (LVEDD), and (**F**) LV end-systolic diameter (LVESD). Data are means ± SEM (n = 10-16 mice per group for day 28). (**G**) Representative images of LV sections stained with Masson's trichrome blue to detect fibrosis. Scale bar, 20 µm. (**H**) Quantification of percentage area occupied by fibrotic tissue. Data are means ± SEM (n = 10-16 mice per group). Analysis by one-way ANOVA and Dunnett's post-hoc test. *$P < 0.05$, **$P < 0.01$, ***$P < 0.001$, ****$P < 0.0001$.

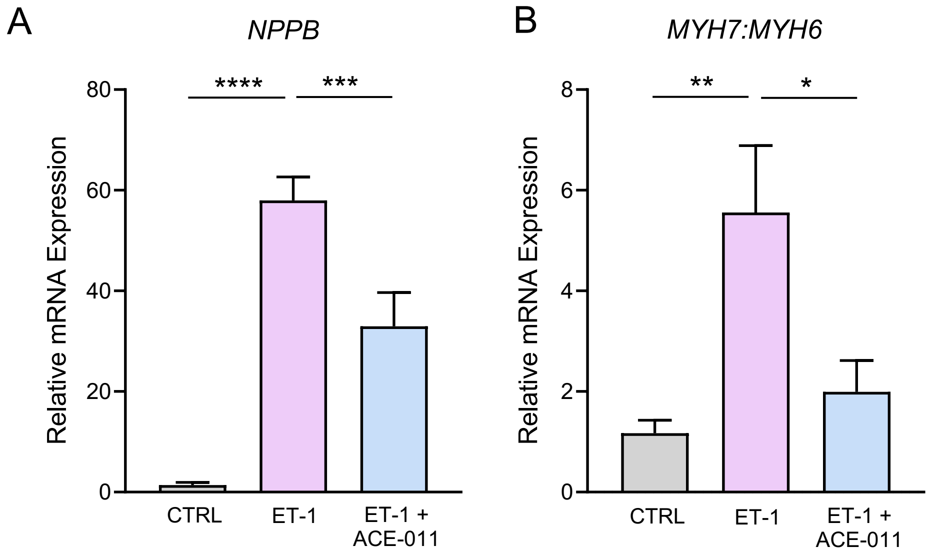

Supplementary Figure 2. ActRIIA-Fc reduces cellular injury in cardiomyocytes derived from human induced pluripotent stem cells. Effects of human ActRIIA-Fc (ACE-011) on endothelin-1 (ET-1)-induced increases in *NPPB* expression (**A**) and *MYH7*:*MYH6* expression ratio (**B**) in human iPSC-derived cardiomyocytes. Data are means ± SEM; n = 4-5 replicates per group. Analysis by one-way ANOVA and Dunnett's post-hoc test. *$P < 0.05$, **$P < 0.01$, ***$P < 0.001$, ****$P <$ 0.0001. CTRL, control.

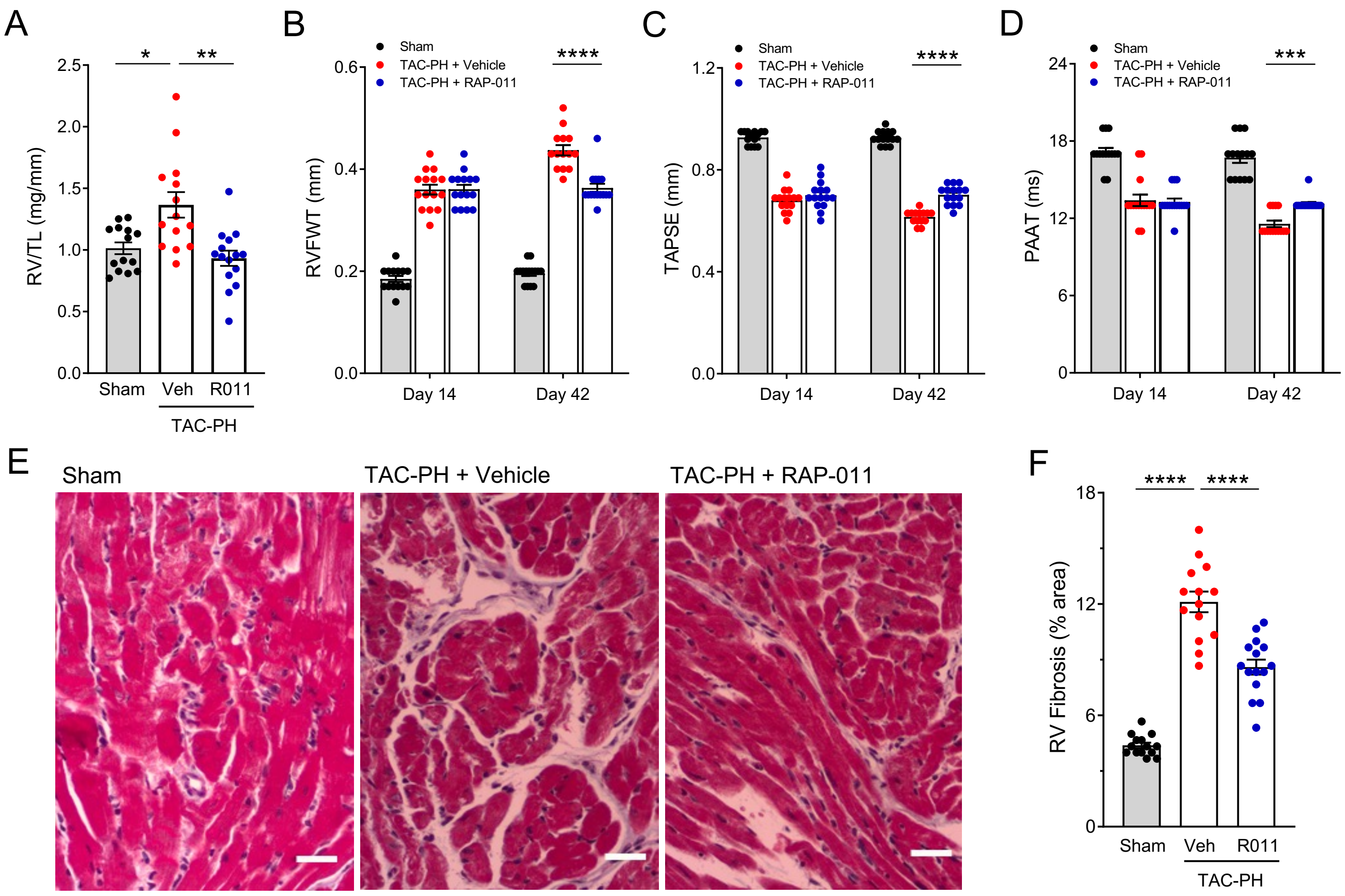

Supplementary Figure 3. ActRIIA-Fc reduces RV remodeling and improves right heart function in a TAC-PH mouse model. (**A**) RV mass normalized to tibia length (RV/TL), (**B**) RV free wall thickness (RVFWT), (**C**) tricuspid annular plane systolic excursion (TAPSE), and (**D**) pulmonary artery acceleration time (PAAT). Data are means ± SEM (n = 10-15 mice per group for day 42). (**E**) Representative images of RV sections stained with Masson's trichrome blue to detect fibrosis (scale bar, 50 µm), and (**F**) quantification of percentage area occupied by fibrotic tissue. Data are means ± SEM (n = 10-15 mice per group). Analysis by one-way ANOVA and Dunnett's post-hoc test. *$P < 0.05$, **$P < 0.01$, ***$P < 0.001$, ****$P < 0.0001$. R011, RAP-011; veh, vehicle (PBS).

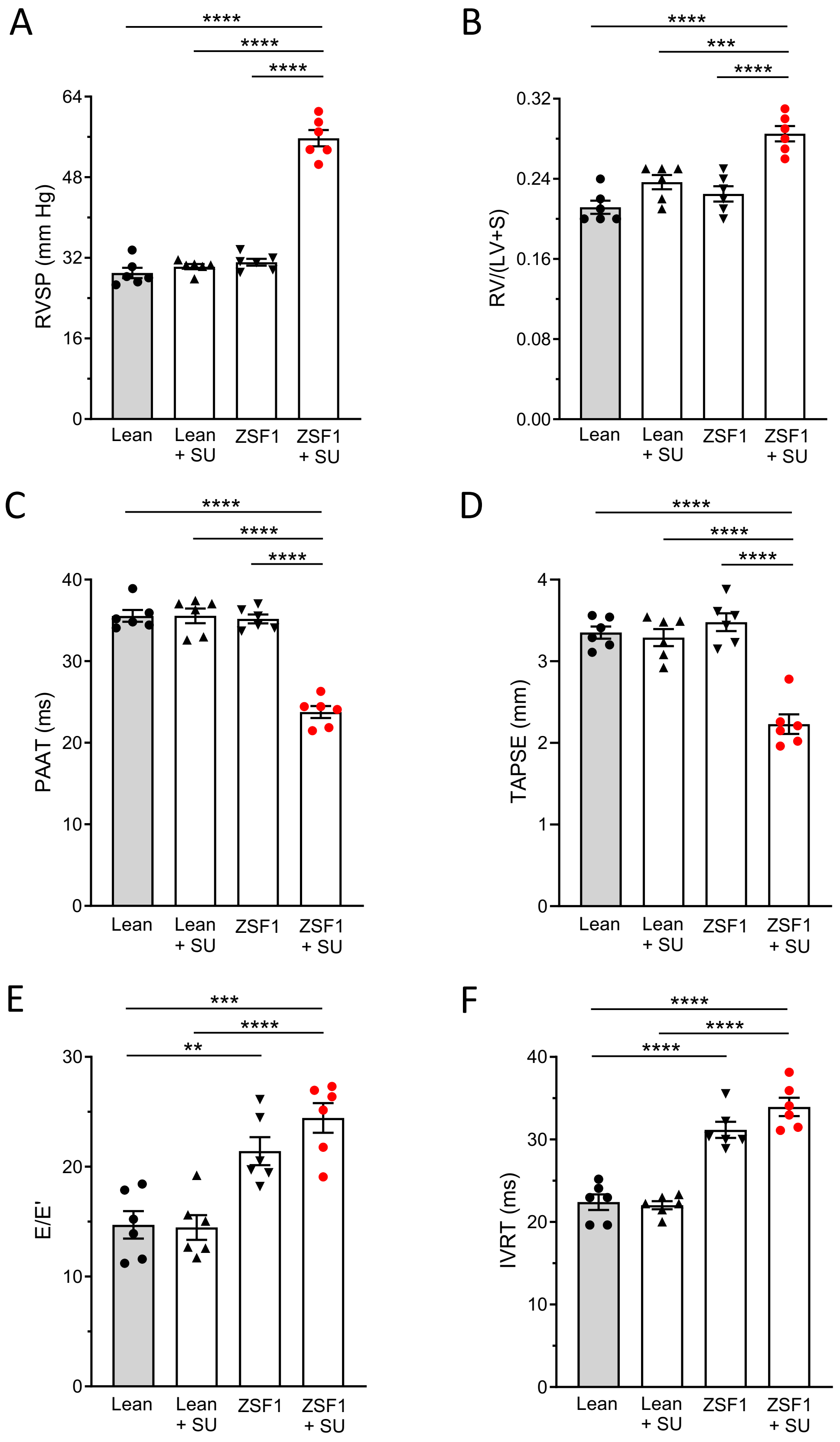

Supplementary Figure 4. A two-hit model of PH-HFpEF induced by SU5416 in obese ZSF1 rats but not lean rats. Obese ZSF1 and lean rats were treated at 8 weeks of age with a single dose of SU5416 (SU, 100 mg/kg, s.c.), an inhibitor of vascular endothelial growth factor receptor, and evaluated after 14 weeks. (**A**) RV systolic pressure (RVSP), (**B**) Fulton index, (**C**) pulmonary artery acceleration time (PAAT), (**D**) tricuspid annular plane systolic excursion (TAPSE), (**E**) ratio of mitral inflow velocity to mitral annular velocity (E/E'), and (**F**) isovolumetric relaxation time (IVRT). Data are means ± SEM. Analysis by one-way ANOVA and Tukey's post-hoc test. **, $P < 0.01$; ***, $P < 0.001$; ****, $P < 0.0001$.

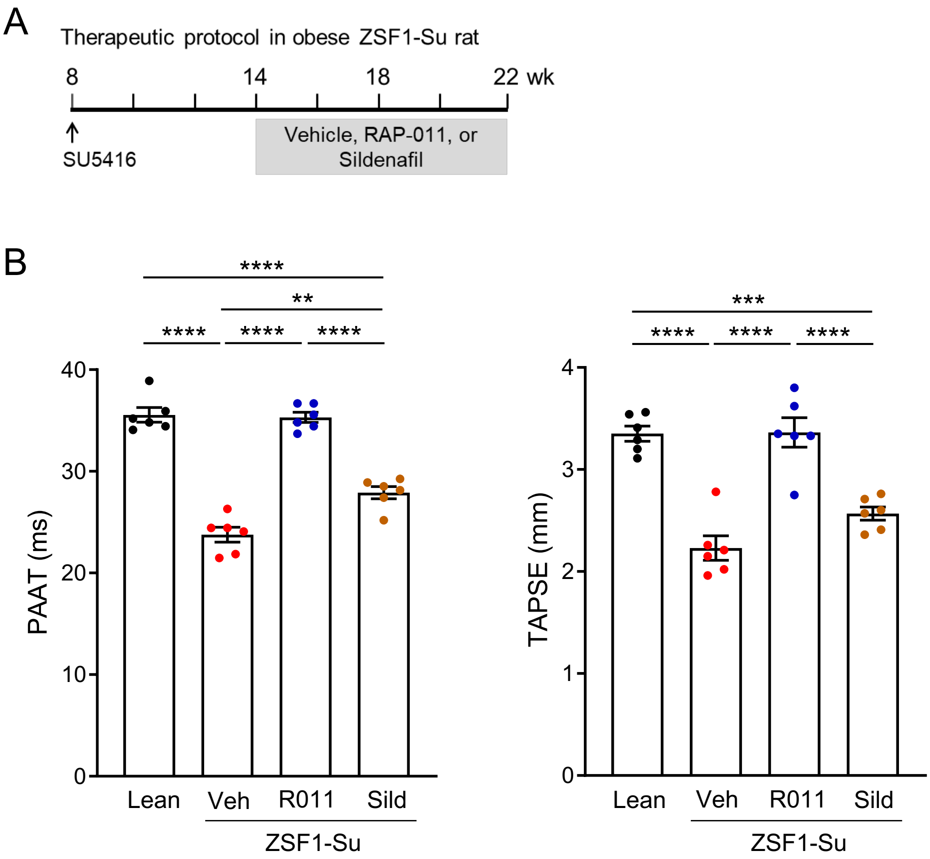

Supplementary Figure 5. Therapeutic treatment with ActRIIA-Fc improves right heart function more effectively than sildenafil in obese ZSF1-Su rats. (**A**) Experimental approach used to evaluate therapeutic effects of ActRIIA-Fc (RAP-011) in an obese ZSF1-Su rat model of PH-HFpEF as in Figure 1A. (**B**) Effects of RAP-011 (R011) or sildenafil (Sild) on pulmonary artery acceleration time (PAAT) and tricuspid annular plane systolic excursion (TAPSE). Data are means ± SEM. Analysis by one-way ANOVA and Tukey's post-hoc test. **, $P < 0.01$; ***, $P < 0.001$; ****, $P < 0.0001$. Lean, lean control rats; veh, vehicle (PBS).

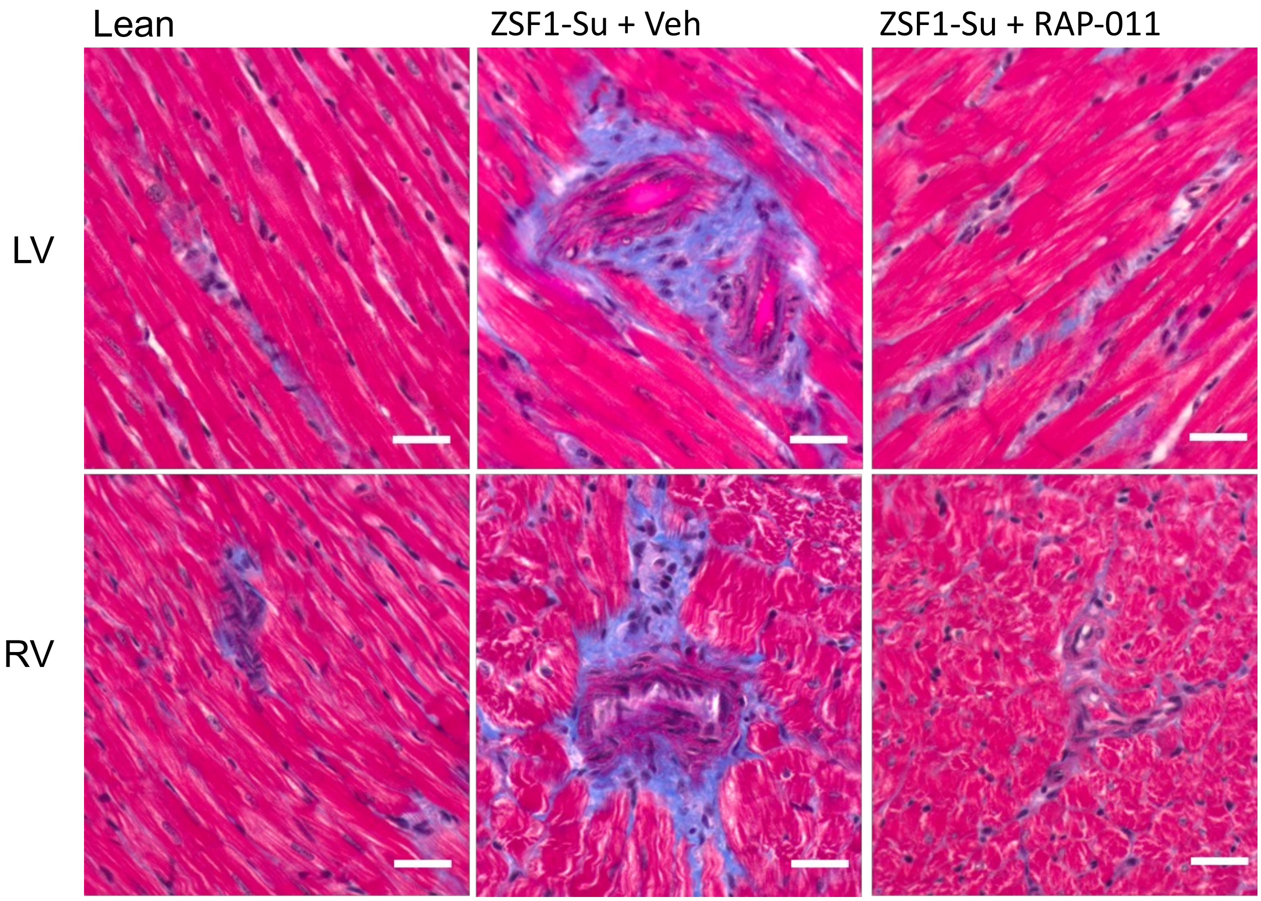

Supplementary Figure 6. ActRIIA-Fc reduces fibrosis in the left and right ventricles in obese ZSF1-Su rats. Rats were treated as in Figure 4A, and representative images are shown from sections of the left ventricle (LV) and right ventricle (RV) stained with Masson's trichrome to detect fibrosis. Veh, vehicle (PBS). Scale bar, 20 µm.

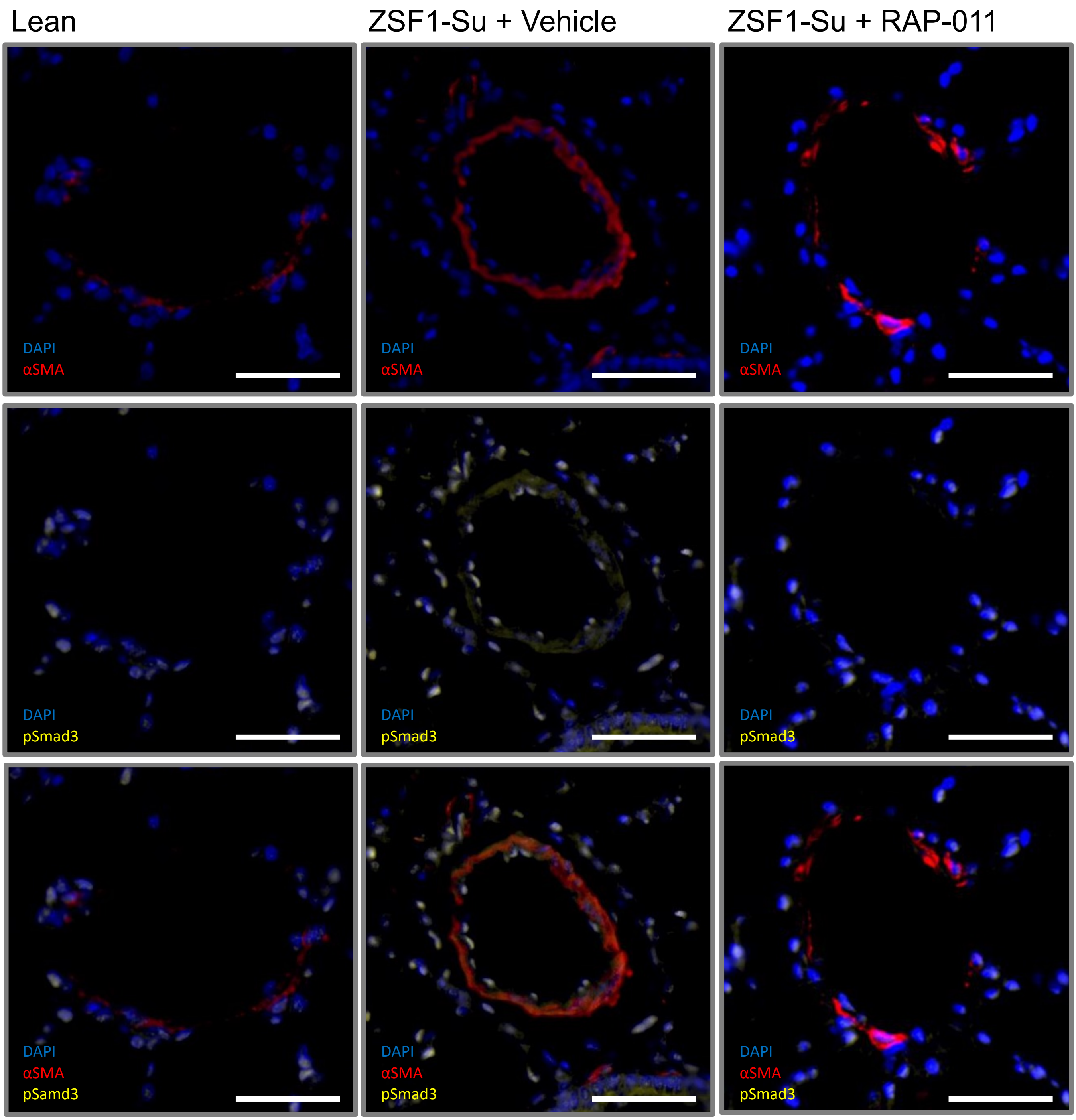

Supplementary Figure 7. ActRIIA-Fc reduces SMAD3 overactivation in pulmonary vascular smooth muscle cells of obese ZSF1-Su rats. Representative immunofluorescence images showing degree of costaining of pSMAD3 and smooth muscle α-actin (αSMA) in paraffin-embedded lung sections from lean rats and ZSF1-Su rats treated with RAP-011 or vehicle (n = 4 per group). DAPI identifies cell nuclei. Scale bar, 50 µm.

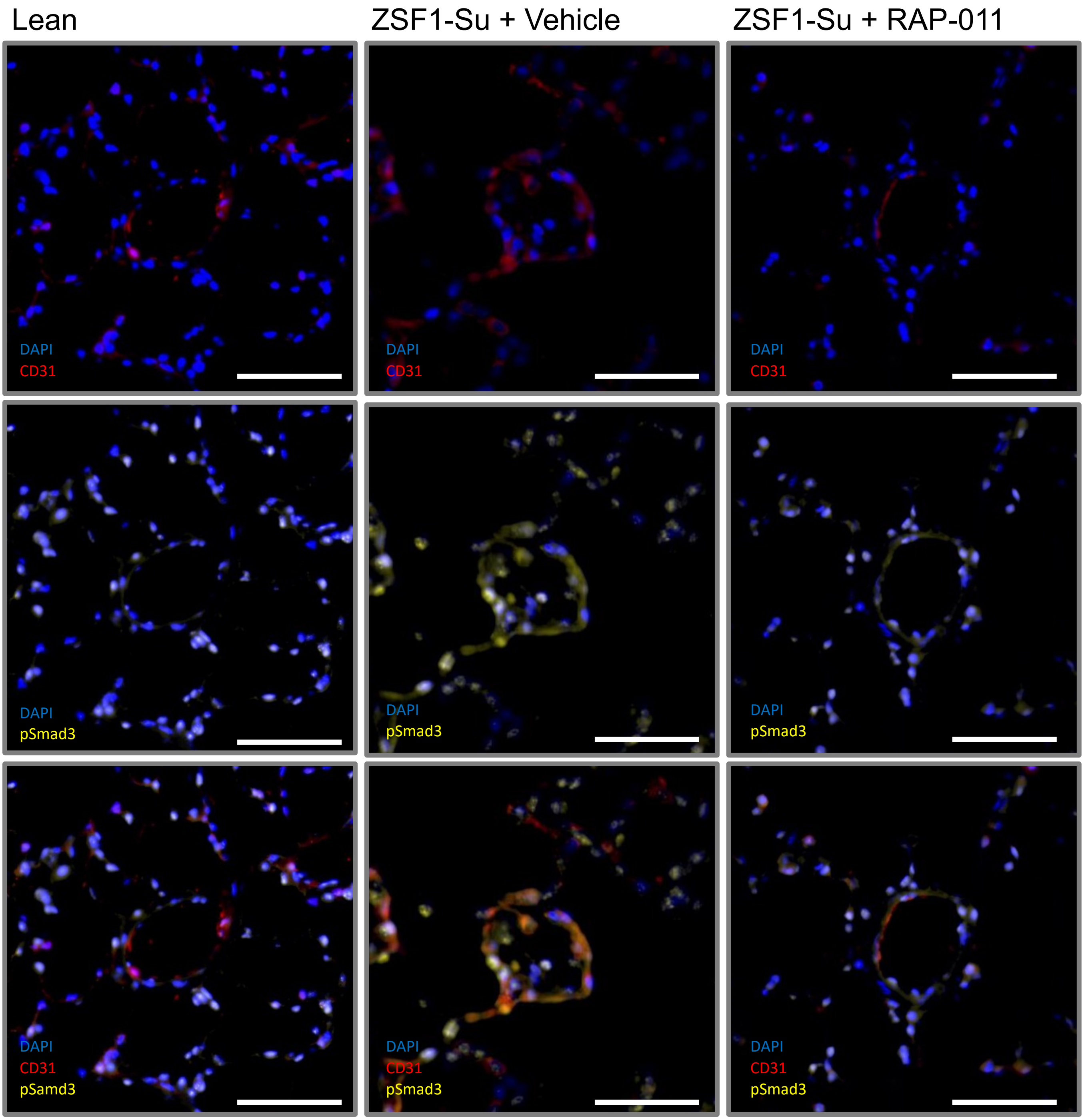

Supplementary Figure 8. ActRIIA-Fc reduces SMAD3 overactivation in pulmonary endothelial cells of obese ZSF1-Su rats. Representative immunofluorescence images showing degree of costaining of pSMAD3 and endothelial cell marker CD31 in paraffin-embedded lung sections from lean rats and ZSF1-Su rats treated with RAP-011 or vehicle (n = 4 per group). DAPI identifies cell nuclei. Scale bar, 50 μm.

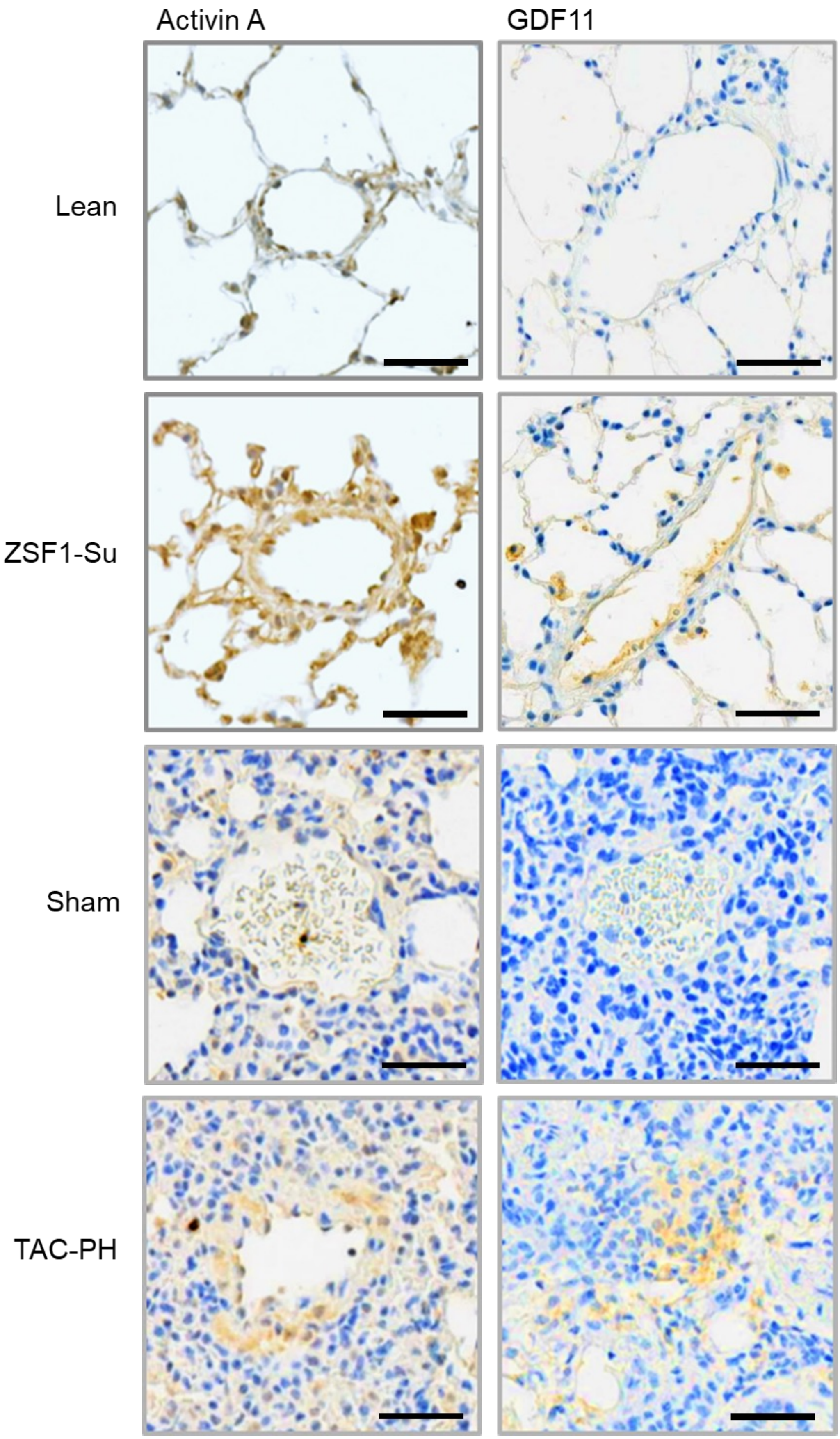

Supplementary Figure 9. Increased expression of activin A and GDF11 in the pulmonary vasculature of PH-LHD models. Representative images of immunohistochemical staining (brown) for activin A and GDF11 in paraffin-embedded lung sections from ZSF1-Su rats and TAC-PH mice (n = 3 per group). Scale bar, 50 µm.
